# Atogepant modulates brain connectivity in episodic migraine: a longitudinal exploratory fMRI study

**DOI:** 10.3389/fneur.2026.1824731

**Published:** 2026-08-25

**Authors:** Danilo Antonio Montisano, Davide Fedeli, Greta Demichelis, Giuseppe Ciullo, Jean Paul Medina Carrion, Emilio Ciusani, Alessandra Erbetta, Marina Grisoli, Alessia Marcassoli, Giulia Regonesi, Alessandra Parisi, Alberto Raggi, Anna Nigri, Licia Grazzi

**Affiliations:** 1 Headache Center, Fondazione IRCCS Istituto Neurologico Carlo Besta, Milano, Italy; 2 Neuroradiology Unit, Fondazione IRCCS Istituto Neurologico Carlo Besta, Milan, Italy; 3 Department of Medicine and Surgery, University of Parma, Parma, Italy; 4 Department of Diagnostic and Technology, Fondazione IRCCS Istituto Neurologico Carlo Besta, Milano, Italy; 5 Neurology, Public Health and Disability Unit, Fondazione IRCCS Istituto Neurologico Carlo Besta, Milano, Italy; 6 Neurology Unit, ASST Bergamo Est, Seriate, Italy

**Keywords:** atogepant, functional connectivity, migraine, neurotransmitters, resting-state fMRI

## Abstract

**Aim:**

Episodic Migraine (EM) is a highly prevalent and disabling neurological disorder. Recent therapeutic advances have targeted the calcitonin gene-related peptide (CGRP), a neuropeptide implicated in the pathophysiology of migraine. Promising outcomes have been obtained with monoclonal antibodies and, more recently, with a new class of drugs consisting of small-molecule CGRP receptor antagonists, known as gepants. Atogepant, an oral agent of the gepant class, has demonstrated efficacy in migraine prevention, yet its effects on central brain activity and neurotransmitter circuits remain largely unclear. This exploratory study investigates changes in brain functional connectivity and changes related to key neurotransmitter systems, following 12 weeks of treatment with atogepant in a group of EM patients.

**Methods:**

This is an exploratory single-arm longitudinal study assessing clinical and neuroimaging changes before and after 12 weeks of atogepant treatment. We enrolled patients diagnosed with EM according to ICHD-3 criteria, without prior exposure to anti-CGRP therapies. Participants underwent clinical assessments (monthly migraine days MMD, acute drugs intake MAM) and resting-state functional MRI (rs-fMRI) before and after 12 weeks of treatment with atogepant 60 mg once daily. Longitudinal functional connectivity analyses were performed at the whole-brain level with a region-of-interest analysis. Additionally, longitudinal neurotransmitter-related functional connectivity was investigated within the serotoninergic and the dopaminergic systems.

**Results:**

A total of 15 patients completed the evaluation. A significant group-level reduction in MMD (T = −7.09, *p* < 0.001) and MAM (T = −6.35, *p* < 0.001) following 12 weeks of atogepant treatment was observed, accompanied by a reduction of allodynia symptoms, albeit not statistically significant. Patients exhibited significant longitudinally increased functional connectivity, involving the right superior frontal gyrus, bilateral putamen, left pallidum, left anterior and bilateral posterior cingulate cortex. The greater the longitudinal increase in anterior–posterior cingulate cortices connectivity, the larger the improvement in MMD, MAM, and allodynia symptoms after treatment. Additionally, longitudinal connectivity changes were observed within the orbitofrontal cortex in the mesocorticolimbic dopaminergic system.

**Discussion:**

In this exploratory cohort, significant clinical improvement after atogepant treatment was accompanied by longitudinal functional connectivity changes in EM patients. These preliminary findings may reflect either direct or indirect central modulation linked to atogepant treatment. A deeper understanding of the observed central changes may help to clarify the mechanisms underlying anti-CGRP therapies for migraine.

## Highlights

- The longitudinal effects of 12 weeks of daily anti-CGRP (atogepant 60 mg) treatment on brain functional connectivity was investigated in 15 patients with episodic migraine.
- Atogepant treatment was associated with significant clinical improvement.
- Longitudinal fMRI analyses revealed increased functional connectivity involving frontal, cingulate, and striatal regions.
- Longitudinal cingulate functional connectivity changes correlated with clinical improvements.
- Dopaminergic mesocorticolimbic connectivity in frontal region increased after atogepant treatment.

## 1 Introduction

Migraine is a common disabling neurological condition, characterized by severe recurrent headaches, accompanied by several neurological and autonomic symptoms (1). Its pathophysiology involves peripheral nociceptive signals and complex central alterations from the trigemino-vascular system, neurotransmitter systems, and central pain modulation regions (1).

Some neuroimaging studies have provided evidence of central dysfunction in patients with migraine, reporting pain-induced brain alterations and suggesting a dysregulation of pain facilitation and inhibition processes (2). Resting-state fMRI (rs-fMRI) studies have further revealed widespread disruptions in functional connectivity (FC) across multiple cortical and subcortical regions and networks implicated in sensory, cognitive, and affective aspects of pain (3–6).

Novel migraine preventive treatments acting on Calcitonin Gene Related Peptide (CGRP) pathway have shown remarkable efficacy in clinical practice (7). CGRP plays a major role in migraine pathophysiology by promoting meningeal vessels dilation, local neurogenic inflammation, thereby releasing pro-nociceptive mediators that enhance pain transmission and sensitivity. Two main classes of CGRP-targeting treatments are available: monoclonal antibodies (mAbs anti-CGRP; e.g., erenumab, galcanezumab) directed against CGRP or its receptor, and small-molecule CGRP receptor antagonists, gepants (7). In this context, atogepant, a recently approved gepant therapeutic drug, has demonstrated high safety (with no significant risk of hepatotoxicity) (8), tolerability, and efficacy in migraine prevention. Clinical evidence shows a rapid and robust preventive effect, with 60–80% of patients achieving a ≥ 50% reduction in monthly migraine episodes within 3 months, and up to ~50% reaching complete remission at 1 year (9–11).

Only a few neuroimaging studies have investigated whether anti-CGRP mAbs could modify brain activity. For erenumab, Schwedt et al. showed changes in network FC efficiency and central processing of extracranial painful stimuli involving cingulate and frontal cortices, putamen, and periaqueductal gray in treatment responders (12). Moreover, placebo-controlled trials showed FC modulation after treatment in visual, cerebellar, and thalamic regions, and decreased subcortical activity during a trigeminal nociceptive fMRI task (13, 14). In patients treated with galcanezumab, responders exhibited reduced FC between the somatosensory, motor, and insular cortices (15), along with a more pronounced decrease in hypothalamic activation (16). By contrast, no neuroimaging evidence is available for gepants. Whereas mAbs act mainly peripherally and have limited blood–brain barrier (BBB) permeability (17), the degree of BBB penetration by gepants is still unclear, and this leaves open the question of its potential central neurofunctional involvement. Moreover, since migraine pathophysiology involves multiple neurotransmitter systems, including serotonin, which contributes to migraine generation, and dopamine, which has been associated with prodromal symptoms such as yawning and nausea, understanding how anti-CGRP treatments modulate neurotransmitter activity may provide new insights into their central mechanisms of action (18, 19).

On these grounds, this exploratory longitudinal study aimed to investigate the changes over a 12-week atogepant treatment on resting state brain FC and on FC modulated by the spatial distribution of different neurotransmitter receptors in a cohort of episodic migraine (EM). To our knowledge, no prior rs-fMRI study has explored the longitudinal effects of atogepant treatment on brain FC.

## 2 Methods

### 2.1 Subjects and clinical assessment

We conducted a longitudinal exploratory study including patients with EM who were naïve to anti-CGRP therapies. Participants underwent clinical and resting-state fMRI assessments at baseline and after 12 weeks of continuous treatment with atogepant 60 mg/day.

Patients with a diagnosis of EM were recruited at our headache center at Fondazione IRCCS Istituto Neurologico Carlo Besta, Milan, Italy. Inclusion criteria were: age > 18 and diagnosis of EM according to the ICHD-3 (less than 15 total headache days per month, and less than 8 migraine days per month) (20). Patients with prior exposure to anti-CGRP therapies, other ongoing preventive therapies, medication overuse headache at enrolment, comorbidity with neurological, cardiovascular, psychiatric disease or diabetes, and pregnant women were excluded. As rescue medication, non-steroidal anti-inflammatory drugs, analgesics, and triptans were allowed. Given the exploratory nature of the study, no formal *a priori* sample-size calculation was performed. Included patients underwent a 12-week treatment with daily atogepant 60 mg, in line with usual recommendations (9). The study timeline was structured as follows. At baseline (T0), clinical assessment was performed, and MRI scans were acquired within 1–4 days. Treatment was then initiated and maintained for 12 weeks, after which a new clinical follow-up (T1) and MRI acquisition (within 1–4 days) were performed. Patients were enrolled from May 2024 to December 2024. Adherence to daily treatment was assessed at T1 using medication blister pack inspection and through direct patient interviews regarding missed doses. The study was approved by the local Ethical Committee (approval CET 48/24 BST 61/23) and all participants provided their consent to the study. For each patient, age, sex, monthly migraine days (MMD) and monthly acute medication (MAM) both assessed with migraine diaries based on average values on the previous 3 months, allodynia (ASC-12), and duration of disease were collected during clinical assessments.

### 2.2 MRI acquisition

Patients underwent MRI acquisition, including a high-resolution structural 3D T1-weighted (T1w) image (Repetition Time [TR]  = 8.11 ms; Echo Time [TE] = 3.71 ms; Field-of-View [FOV]  = 240 × 240 mm; voxel size = 1 × 1 × 1 mm^3^; flip angle = 8°; 185 sagittal slices) and a rs-fMRI scan (15 min, eyes-opened, with a black screen; T2*-weighted BOLD echo-planar imaging gradient-echo sequence; TR = 2000 ms; TE = 30 ms; FOV = 80 × 80 mm; voxel size = 3 × 3 × 3.2 mm^3^; interslice gap = 0.4 mm; flip angle = 80°; 34 axial slices; 450 volumes; Phase Encoding direction = posterior/anterior), using a 3 T Philips Achieva-dStream scanner, with a 32-channel head coil. The eyes open rs-fMRI acquisition was monitored with an integrated camera system during rs-fMRI acquisition. Moreover, patients were interviewed immediately after each session to confirm they were awake during the scan. Patients were headache-free for at least 24 h prior to and during the MRI acquisitions. The pain-free state was systematically assessed by a physician through patients’ clinical interview before and after each MRI session. All images were inspected by an expert neuroradiologist (A. E.) to exclude brain abnormalities or artefacts affecting the exam quality.

### 2.3 Rs-fMRI pre-processing

Image quality control of rs-fMRI scan was performed using MRIQC software (21). Rs-fMRI data were analyzed with the CONN toolbox (v. 20.b; running on Matlab 2020b) (22) using the default-MNI pipeline. For each patient and each session, functional volumes were realigned to the first volume, unwrapped, and slice-time corrected. The ART toolbox, implemented within CONN, was used for outlier volumes identification and scrubbing (framewise displacement>0.9 mm; global BOLD signal changes >5 SD; default CONN settings). Both rs-fMRI and T1w images were normalized to the MNI space and functional images were smoothed with a 6 mm full width at half-maximum (FWHM) gaussian kernel. Rs-fMRI data were denoised using the anatomical component-based noise correction, with white matter and cerebrospinal fluid components derived from T1w image segmentation. The effects of realignment parameters from motion correction, outlier volumes scrubbing parameters, physiological noise components, and initial magnetization transient effects were regressed out. Data were then bandpass-filtered with a 0.008–0.1 Hz filter.

### 2.4 Neurotransmitter-related functional connectivity pre-processing

To investigate the FC in relation to the spatial distribution of underlying neurotransmitter systems, we employed the Receptor-Enriched Analysis of functional Connectivity by Targets (REACT) approach. REACT-fMRI toolbox^1^ (23) employs a two-step regression approach (also referred to as a “dual regression”) to estimate subject-specific FC maps guided by the distribution of neurotransmitters, using publicly available molecular templates generated from PET scans of healthy individuals (24, 25). Importantly, REACT identifies patterns of resting-state FC informed by receptor and transporter density maps, rather than directly measuring neurotransmission (e.g., neurotransmitter release, or receptor occupancy). Reference molecular maps from serotonin and dopamine were used to generate neurotransmitter-enriched FC maps. The serotonin atlas included maps of 5-HT_1a_, 5-HT_1b_, 5-HT_2a_, 5-HT_4_ receptors, and the serotonin transporter (5-HTT). The dopamine atlas included maps of D_1_ and D_2_ receptors, and the dopamine transporter (DAT). The cerebellum was removed from the serotonin atlas and the D_1_ and D_2_ templates, while the occipital cortex was removed from the DAT map, as they were used as reference regions in radioligands kinetic models (24, 25). Intensity values were then normalized (i.e., 0 minimum to 1 maximum) for each image.

FC maps informed by serotonin maps were masked with the standard MNI gray matter binary mask provided within REACT. Dopamine maps were further subdivided into two pathways: mesocorticolimbic (MCL) and nigrostriatal (NS) systems. Two binary gray matter masks were created to better characterize these pathways, as recently described in Manca et al. (26). The MCL mask included the following cortical and subcortical regions: anterior cingulate cortex (ACC), amygdala, hippocampus, parahippocampus, inferior frontal orbital cortex, entorhinal cortex, subcallosal gyrus, nucleus accumbens, lateral frontal orbital cortex, middle frontal gyrus, medial frontal gyrus, superior frontal gyrus (SFG) from WFU PickAtlas toolbox,^2^ as well as the ventral tegmental area (27); the NS mask included caudate nucleus, substantia nigra, putamen and globus pallidus from WFU PickAtlas toolbox.

### 2.5 Statistical analyses

#### 2.5.1 Demographic and clinical assessment

Normality of clinical variables was assessed using the Kolmogorov–Smirnov test. Within-subject longitudinal differences in these variables were assessed using paired-sample t-tests (or the corresponding nonparametric equivalent in case normality was not satisfied). All analyses were performed in Python.

#### 2.5.2 Rs-fMRI analysis

To explore longitudinal changes in FC aftera 12-week atogepant treatment in EM patients, we performed a longitudinal region of interest (ROI)-to-ROI analysis in CONN toolbox. For each participant and each session, a connectivity matrix including all cortical and subcortical ROIs from the Hammersmith atlas (28–31) was computed. Pearson correlation coefficients were calculated between the time series of each ROI and those of all other ROIs. These correlation values were subsequently transformed into Z scores using the Fisher R-to-Z transformation, resulting in a whole-brain connectivity matrix for each participant and time point. These matrices were then analyzed using a second-level general linear model to assess longitudinal changes in network connectivity pre- and post-treatment. Age and sex were not entered as covariates as they are intrinsically controlled in within-subject analyses. A connection-level uncorrected threshold of *p* < 0.05 was used to identify the strongest individual connections (as per CONN’s default settings in Functional Network Connectivity ROI-to-ROI analyses). Only ROI-to-ROI connections that met this criterion were retained for further analysis. Additionally, to correct for multiple comparisons and control the false discovery rate, a cluster-level False Discovery Rate (FDR) threshold of *p* < 0.05 was applied to the significant clusters (i.e., groups of similar connections within the ROI-to-ROI graph).

#### 2.5.3 Neurotransmitter-related functional connectivity analysis

To explore changes in FC in relation to the spatial distribution of neurotransmitters, after the 12-week atogepant treatment, longitudinal neurotransmitter-related FC maps were generated for each subject and each neurotransmitter of interest by subtracting the T0 maps from the corresponding T1 map. These difference maps were then entered into a set of separate second-level general linear models using SPM12. For each model, results were thresholded with a *p* < 0.001 uncorrected at the voxel level and Family-wise Error Rate (FWE, SPM’s default correction) corrected for multiple comparisons at *p* < 0.05 at the cluster level.

#### 2.5.4 Correlation between rs-fMRI connectivity and clinical scales

Longitudinal differences in FC values from significant ROI-to-ROI connections (i.e., connectivity at T1 minus connectivity at T0) and neurotransmitter-related FC of significant clusters obtained with REACT were correlated with longitudinal differences in clinical variables, such as MMD, MAM and allodynia scores (i.e ΔMMD, ΔMAM, ΔASC-12). Outlier detection was performed with Interquartile Range method. Significance was set at *p* ≤ 0.05 uncorrected. All analyses were performed in Python. Given the exploratory nature of the study and the small sample size, these correlation analyses were intended only as preliminary observations. Therefore, results should be interpreted with caution.

## 3 Results

### 3.1 Subjects and clinical data

A total of 17 EM patients was enrolled. Two patients did not complete the program due to MRI intolerance, leaving a total of 15 patients (Table 1). Prior preventive failure lines included antidepressants (10 occasions), antiepileptic drugs (6 occasions), beta-blockers (5 occasions), botox (4 occasions), ca-antagonist (5 occasions). The following comorbidities beyond major exclusion criteria were reported: recurrent urinary tract infection (2), previous genecology surgery for benign lesions (2), glaucoma (1), obesity (2), hypercholesterolemia (1). No patient was excluded due to excessive motion or artefacts during MRI acquisition. Patients scrubbed/valid scans ratio was similar between sessions (T = 0.498, *p* = 0.626). All patients were classified as EM without aura except for one, with EM without aura and sporadic migraine with visual aura. Patients showed a significant longitudinal decrease in MMD and MAM. Similarly, allodynia scores decreased at the follow-up, although the change did not reach statistical significance (see Table 1).

**Table 1:** Demographic and clinical data (median ± standard deviations, range).

Variable	T0	T1	T-value; *p*-value
Sex (F/M)	13/2	--	--
Age at T0 (years)	43 ± 15.3 (19–67)	--	--
Age of Onset (years)	15.79 ± 9.7 (6–40)	--	--
Disease Duration (years)	26.14 ± 15.1 (8–59)	--	--
MMD	10 ± 2.3 (5–13)	2 ± 2.8 (0–10)	−7.09; <0.001
MAM	12 ± 3 (5–15)	2 ± 3.6 (0–13)	−6.35; <0.001
ASC-12	4 ± 4.1 (0–14)	0.5 ± 6.20 (0–19)	−0.51, 0.62

The results of within-subjects t-test are reported. F/M, Females/Males; T0, baseline; T1, follow-up after 12 weeks of atogepant treatment; MMD, monthly migraine days; MAM, acute drugs intake; ASC-12, allodynia score.

### 3.2 Rs-fMRI analysis

**Figure 1:**
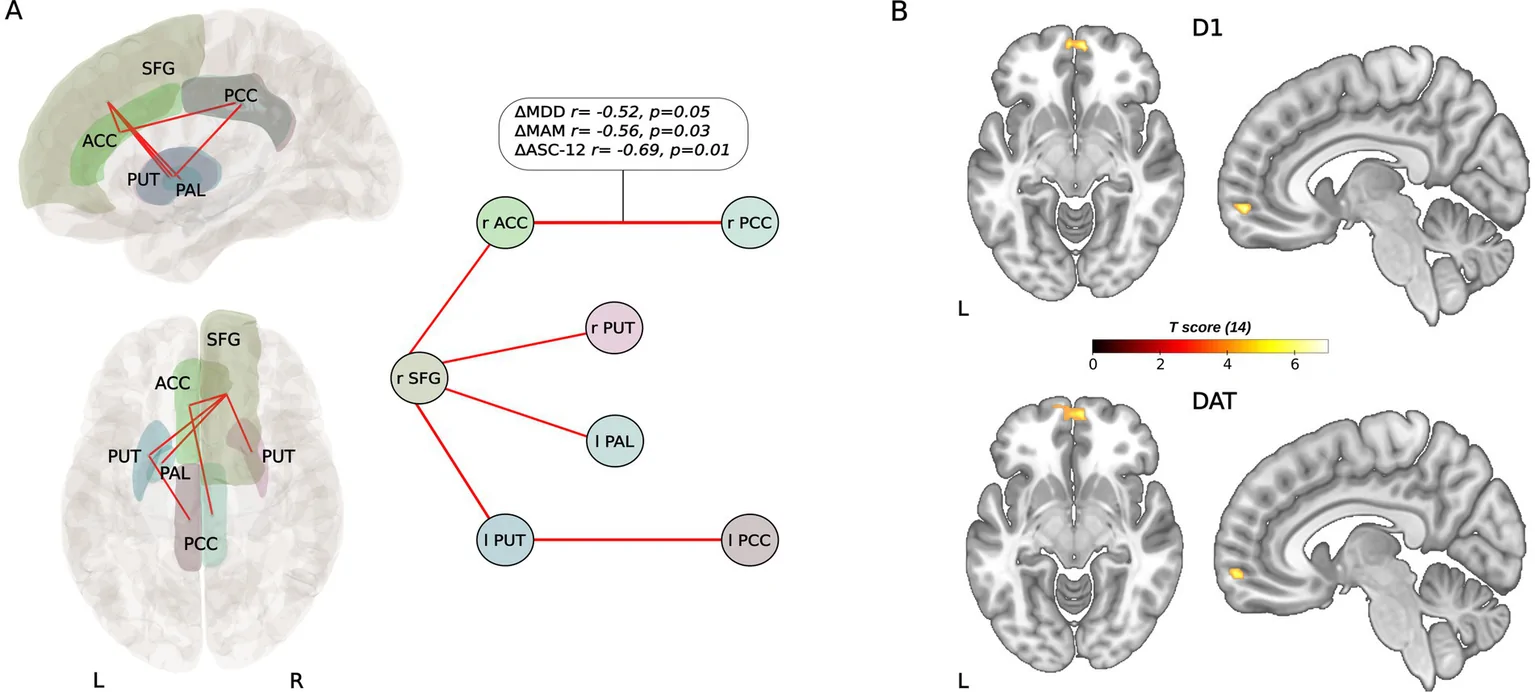
**(A)** Shows longitudinally increased region-of-interest to region-of-interest functional connectivity changes after 12 weeks of atogepant treatment. All results are reported at *p* < 0.05 FDR-corrected at the cluster level and *p* < 0.05 uncorrected at the connection level. Results are reported in sagittal view, axial view, and as a connectivity graph. Correlations between connectivity differences and clinical improvement are reported in the connectivity graph. **(B)** Shows longitudinally increased neurotransmitter-related functional connectivity changes after 12 weeks of atogepant treatment in the mesocorticolimbic dopaminergic system for D1 receptor and DAT transporter. All results are reported at *p* < 0.05 FWE-corrected at the cluster level and *p* < 0.001 uncorrected at the voxel level. Axial slices are reported at MNI z = −8; sagittal slices are reported at MNI x = 6. SFG, Superior Frontal Gyrus; ACC, Anterior Cingulate Cortex; PCC, Posterior Cingulate Cortex; PUT, Putamen; PAL, Globus Pallidus; r/R, right hemisphere; l/L, left hemisphere; Δ, longitudinal difference (treatment vs. baseline); MMD, monthly migraine days; MAM, acute drugs intake; ASC12, allodynia score.

Longitudinal ROI-to-ROI analyses revealed that atogepant treatment was accompanied by significant connectivity changes in frontal, cingulate, and subcortical regions. Specifically, we observed increased FC of the rSFG with the bilateral putamen, the left pallidum, and the left ACC. Increased connectivity was also observed between the left PCC and the left putamen, and between the right PCC and the left ACC (Table 2; Figure 1A).

**Table 2:** Longitudinal ROI-to-ROI analyses.

Region 1	MNI xyz coordinates	Region 2	MNI xyz coordinates	T-value	*p*-unc
Superior frontal gyrus R	13,32,36	Putamen R	25,4,0	3.54	0.003**
Superior frontal gyrus R	13,32,36	Putamen L	−26,2,0	3.17	0.007**
Superior frontal gyrus R	13,32,36	Pallidum L	−20,−2,−1	3	0.009**
Superior frontal gyrus R	13,32,36	Anterior cingulate gyrus L	−6,27,21	2.76	0.012*
Posterior cingulate gyrus L	−5,−30,35	Putamen L	−26,2,0	2.31	0.037*
Posterior cingulate gyrus R	6,−27,35	Anterior cingulate gyrus L	−6,27,21	2.15	0.049*

Results of region-of-interest to region-of-interest analyses of functional connectivity changes after 12 weeks of atogepant treatment are reported. (cluster statistics: F=19.5; *p*-FDR = 0.046). All results are thresholded at *p* < 0.05 uncorrected (*p*-unc) at the connection level and *p* < 0.05 FDR-corrected at the cluster level. *p < 0.05; ** *p* < 0.01. R, right hemisphere; L, left hemisphere.

### 3.3 REACT analysis

Longitudinal neurotransmitter-related analyses revealed that atogepant treatment was accompanied by significant changes in dopaminergic-related FC. Following 12-week atogepant treatment, patients exhibited increased FC within the MCL dopaminergic system (D1 receptor and DAT transporter), in a cluster located in the medial orbitofrontal cortex (Table 3; Figure 1B).

**Table 3:** Longitudinal neurotransmitter-related functional connectivity analyses.

Neurotransmitter	Receptor/Transporter	Contrast	Region (Hammersmith)	MNI x y z	Cluster (FWE)	K (mm^3^)	T value	Z score	Peak (unc.)	Cohen’s d (95% CI)
Dopamine (MCL)	D1	T1 > T0	R Superior frontal gyrus/Medial orbital gyrus	6,58,−8	0.05	66	6.37	4.3	<0.001	2.47
Dopamine (MCL)	DAT	T1 > T0	R Superior frontal gyrus/Medial orbital gyrus	6,62,−8	0.04	74	5.67	4.02	<0.001	2.35

Results of neurotransmitter-related functional connectivity changes after 12 weeks of atogepant treatment are reported. All results are thresholded at *p* < 0.05 FWE-corrected at the cluster level and *p* < 00.001 uncorrected (unc.) at the voxel level. MCL, mesocorticolimbic system; T0, baseline; T1, follow-up after 12 weeks of atogepant treatment; R, right hemisphere; K, cluster extent; CI, confidence Interval.

### 3.4 Correlation between rs-fMRI connectivity and clinical scales

The correlation analysis of longitudinal changes in ROI-to-ROI FC with longitudinal differences in clinical variables showed a significant association. Greater longitudinal increases in FC between the right PCC and the left ACC were associated with larger reductions over the 12-week treatment period in MMD (ΔMMD; *r* = −0.52, *p* = 0.05), MAM (ΔMAM; *r* = −0.56, *p* = 0.03), and ASC-12 scores (ΔASC-12 *r* = −0.69, *p* = 0.009). No significant correlations were found between longitudinal changes in neurotransmitter-related FC clusters and clinical variables.

## 4 Discussion

In this exploratory study, we observed that 12-week atogepant treatment in EM patients is associated with significant changes in the brain’s FC at rest and dopamine-related FC within the mesocorticolimbic system, accompanied by a significant clinical improvement, in both MMD and MAM, consistent with atogepant efficacy data (11). Furthermore, albeit not significant, we also observed a concomitant decrease in allodynia, a marker of central sensitization. This is partly consistent with previous findings which showed marked decrease in ASC-12 (32, 33), for example with erenumab (34), where this effect was attributed to prolonged suppression of peripheral nociceptive input. Despite the exploratory nature of the present study, which requires considerable caution in interpretation, these longitudinal findings may be compatible with a possible effect of atogepant on central sensitization-related mechanisms. Future studies considering a placebo condition/healthy control group are required to confirm this specific neuromodulatory effect of atogepant.

Longitudinal ROI-to-ROI analyses showed a pattern of increased connectivity at T1, involving the right SFG, bilateral putamen, left pallidum, left ACC, and bilateral PCC. Importantly, the greater the longitudinal increase in ACC-PCC connectivity, the larger the improvement in MMD, MAM, and allodynia scores after treatment, suggesting a possible correspondence between FC modulation and clinical changes. All these regions participate in different aspects of the neural processing of the pain experience, with either pain-facilitating or pain-inhibiting functions and show functional alterations in migraine patients (1–3, 35).

Among all ROIs, the SFG exhibited the largest number of ROI-to-ROI connections showing significant longitudinal changes, highlighting its central involvement in network reconfiguration. This finding should be considered alongside previous data (36) reporting reduced connectivity between the dorsolateral prefrontal cortex and several cortical and basal ganglia regions, including the putamen, in EM patients. Given the critical involvement of SFG in pain modulation, both findings point to a dysfunction of descending pain-control pathways both interictally and in relation to longitudinal reorganization. In this framework, the increased frontal-putaminal connectivity observed after atogepant may potentially reflect a strengthening of interictal fronto-striatal coupling and a reinforcement of top-down pain modulation mechanisms, possibly compatible with a shift of connectivity patterns toward a more balanced network organization.

The SFG also showed longitudinally increased connectivity with the ACC, which in turn exhibited enhanced connectivity with the PCC, suggesting a strengthening of frontal-anterior cingulate-posterior cingulate interactions after treatment. Within this axis, the ACC plays a central role: it integrates the sensory, emotional, and motivational components of the pain experience, contributes to autonomic regulation, and acts as a key node of descending pain-control pathways (37–39). The PCC, on the other hand, is a core hub of the default mode network, involved in the conscious experience of pain (39). Together with the superior frontal cortex, these regions form a network implicated in higher-order modulation of nociceptive processing. Findings from migraine cohorts further highlight the relevance of these midline regions. Consistent structural and functional alterations were reported in the prefrontal cortex, ACC, and PCC (1, 40–43). These functional abnormalities include reduced interictal metabolism in both cingulate regions, particularly in the ACC, where lower metabolic activity correlates with longer disease duration and higher attack frequency (44) as well as altered FC. In particular, stronger coupling between the PCC, ACC, and medial prefrontal regions has been associated with more efficient conditioned pain modulation (45), whereas pain catastrophizing corresponds to reduced activation of these same nodes (46). Resting-state ACC-PCC (47) and PCC (46) connectivity has also been negatively correlated with pain intensity, and patients with cutaneous allodynia exhibit heightened cingulate responses to painful stimulation (47), reinforcing the role of this pathway as critical for endogenous pain regulation and possibly involved in central sensitization in migraine.

In this context, the increase in frontal-ACC-PCC connectivity observed in our cohort, and the ACC-PCC FC association with reductions in MMD, MAM, and allodynia, may indicate longitudinal changes in the functional organization of this midline regulatory system accompanying clinical improvement.

Interestingly, the regions identified in our study (i.e., frontal areas, cingulate cortex, and putamen) overlap with those showing increased connectivity in response to painful stimulation in patients who responded to an 8-week erenumab treatment compared with non-responders (12). Considering that erenumab and atogepant targets both the CGRP receptor, these converging findings raise the possibility of a partially similar, albeit likely indirect, central mechanism of action underlying therapeutic response (38, 40, 43).

With respect to neurotransmitter-related FC, we observed increased dopamine-related FC in the mesocorticolimbic dopaminergic system (D1 receptor and DAT transporter), in a cluster located in the medial orbitofrontal cortex. This region is a key node of the mesocorticolimbic circuit, that contributes to pain modulation by integrating the affective and motivational dimensions of painful stimuli and by exerting inhibitory coupling with regions involved in nociceptive information processing (48). Alterations of this dopaminergic pathway have already been described in migraine, where mesocorticolimbic FC alterations associated with dopamine availability have been previously reported in EM patients, suggesting a dopaminergic contribution to pain processing and affective response to pain (49). Importantly, similar patterns have been observed across chronic pain conditions. Reduced dopamine synthesis and release, together with gray matter volume loss of its major nodes, have been observed (50). Likewise, disrupted mesocorticolimbic connectivity has been associated with central pain sensitization and chronification in conditions such as chronic low back pain (51). Based on these observations, the dopamine-related FC increases observed here may reflect longitudinal changes in mesocorticolimbic dynamics, raising the possibility that modifications in dopamine-related FC patterns may occur during atogepant treatment, possibly accompanying more adaptive pain processing mechanisms. Alternatively, the engagement of the OFC within the mesocorticolimbic system may also relate to reward/relief processes associated with successful treatment in EM patients (i.e., “feeling better”), rather than to a direct pharmacological action on dopaminergic pathways. No significant changes in serotonin-related FC were observed after 12 weeks of atogepant treatment. While dopamine-related FC may have been more directly modulated by treatment, we cannot exclude that changes in FC associated with serotonergic pathways might emerge in larger samples, consistent with the documented role of serotonin in migraine pathophysiology. Although atogepant antagonism of the CGRP receptor is well established, the extent to which atogepant crosses the BBB remains uncertain. Both gepants and mAbs exhibit limited BBB permeability and are therefore believed to exert their therapeutic effects outside the central nervous system (CNS) (52, 53). For mAbs, the prevailing view is that reducing peripheral activation afferents may secondarily influence central brain regions involved in pain perception, salience processing, and descending inhibitory control (12, 13, 15–17, 54). On the other hand, even small quantities reaching the CNS may still contribute to treatment response (17). Although slightly higher in BBB penetration, a similar mechanism is plausible even for gepants.

In this framework, we speculate that the clinical improvements observed in our cohort together with the longitudinal increases in connectivity across regions involved in multiple dimensions of pain processing, including the mesocorticolimbic dopaminergic system, may potentially reflect indirect central modulation associated with atogepant treatment resulting from reduced peripheral nociceptive drive. Given the exploratory nature of this study, and considering the small sample size, further work is needed to clarify whether atogepant, and gepants broadly, exert central actions directly or predominantly through peripheral CGRP-receptor blockade and comparative studies with mAbs will be crucial to clarify the exact mechanisms of anti-CGRP therapies.

### 4.1 Limitations

Our findings should be considered in light of several important limitations. *First*, our sample size of EM patients was small, although clinically homogeneous, reflecting the exploratory nature of the study, and in line with recent MRI studies on anti-CGRP treatment (55). Limited sample sizes increase the risk of false-positive findings, therefore our results should be interpreted with great caution and regarded as preliminary. This limitation is particularly relevant for the correlation analyses, as the observed results may be unstable and require replication in larger cohorts. *Second*, the follow-up period was limited to 12 weeks. While this interval aligns with standard clinical practice, longer observation may help clarify the extent and durability of atogepant CNS effects. *Third*, this cohort study did not include a placebo or untreated control group. Therefore, we cannot determine whether the observed variations in brain connectivity are specifically related to atogepant treatment alone or instead reflect a combination of placebo effects, spontaneous fluctuations, behavioral changes, or other non-specific longitudinal factors. Moreover, other migraine-related variables (e.g., sleep quality, caffeine intake, hormonal status) that might influence resting-state connectivity were not systematically recorded at the time of MRI acquisition. While the within-subject design partially mitigates inter-individual variability and stable intra-individual influences, it does not allow drug-specific effects to be disentangled from these alternative explanations. Future studies in larger samples including a placebo condition or a waiting-list control group and detailed recording of migraine-related factors could further clarify the specific neuromodulatory effects of atogepant.

## 5 Conclusion

In this exploratory study, we report the first preliminary evidence of longitudinal FC changes in brain regions involved in pain processing and within the dopaminergic mesocorticolimbic system in EM patients after 12 weeks of atogepant treatment. While exploratory and requiring cautious interpretation in the absence of a control condition, these findings may reflect direct or indirect central neurofunctional adaptations accompanying clinical improvement. A deeper understanding of such central changes could offer new insights into the mechanism underlying successful preventive therapies for migraine.

## Data Availability

The datasets presented in this article are not readily available however the aggregated data may be made available upon reasonable request to corresponding author. Requests to access the datasets should be directed to Davide Fedeli, davide.fedeli@istituto-besta.it.

## Footnotes

1
https://github.com/ottaviadipasquale/react-fmri

2
https://www.nitrc.org/projects/wfu_pickatlas
